# Microscopic distribution of syringin in freeze‐fixed *Syringa vulgaris* stems

**DOI:** 10.1002/pld3.155

**Published:** 2019-08-02

**Authors:** Dan Aoki, Wakaba Okumura, Takuya Akita, Yasuyuki Matsushita, Masato Yoshida, Yuzou Sano, Kazuhiko Fukushima

**Affiliations:** ^1^ Graduate School of Bioagricultural Sciences Nagoya University Nagoya Japan; ^2^ Research Faculty of Agriculture Hokkaido University Sapporo Japan

**Keywords:** cryo‐TOF‐SIMS, lignification, microscopic distribution, syringin

## Abstract

Monolignols are precursors of lignin, and their glucosides are often found in plants. Glucosylation creates water‐soluble and chemically stable monolignols by protecting the phenolic hydroxyl group. To discuss the role of sinapyl alcohol glucoside, syringin, *in planta*, the cellular distribution of syringin in the transverse and radial surfaces of quick‐frozen stems of *Syringa vulgaris* L. (lilac) was visualized by cryo‐time‐of‐flight secondary ion mass spectrometry and scanning electron microscopy (cryo‐TOF‐SIMS/SEM) analyses. The amount and rough distribution of syringin were confirmed by high‐performance liquid chromatography measurements using serial tangential sections of freeze‐fixed lilac stems. The syringin distribution was also discussed with reference to the tissue classification from microscopic observations. Syringin was mainly found in the phloem region. In the xylem region, syringin was evenly distributed irrespective of the cell type from the cambial zone to the early differentiating stage region and selectively distributed in vessels in the later differentiating stage region. After the lignification of wood fibers, syringin was found in rays and some vessels in the initial part of the annual rings. Previously, artificially administered isotope‐labeled syringin was shown to be assimilated into lignin in the differentiating xylem region. Based on this, our present data showing syringin storage in the differentiating xylem region and its variation depending on the lignification stage suggest that syringin works as a lignin precursor. Additionally, detection of syringin in vessels and rays indicates intercellular transportation of syringin in lilac stems.

## 1 INTRODUCTION

Lignin is an important polymeric compound in plant cell walls. The chemical structure and physicochemical characteristics of lignin have been studied for a long time (Whetten, MacKay, & Sederoff, 1998; Boerjan, 2003; Weng & Chapple, 2010). The main structural units of lignin are phenylpropanoids derived from monolignols. Their structure and composition are different among plant species, tissues, cells, and positions in the cell wall within species. The controlling mechanism of lignin biosynthesis is complicated and remains a controversial topic (Vanholme, Demedts, Morreel, Ralph, & Boerjan, 2010).

The most common structural unit of lignin is the guaiacyl unit derived from coniferyl alcohol. Coniferyl alcohol glucoside, coniferin, is often found in the differentiating xylem region of several plants (Freudenberg & Harkin, 1963; Terazawa, Okuyama, & Miyake, 1984; Savidge, 1989; Fukushima, Taguchi, Matsui, & Yasuda, 1996, 1997), and the role of coniferin in the lignification mechanism has been discussed (Freudenberg, Reznik, Fuchs, & Reichert, 1955; Freudenberg & Harkin, 1963; Dharmawardhana, Ellis, & Carson, 1995; Whetten et al., 1998; Samuels et al., 2002; Terashima, Ko, Matsushita, & Westermark, 2016). Furthermore, in previous experiments, the administration of isotope‐labeled monolignol glucosides to plants showed that these glucosides are assimilated into lignin without altering the natural lignification process (Terashiam & Fukushima, 1988; Fukushima & Terashima, 1990, 1991a, 1991b; Xie, Yasuda, & Terashima, 1994; Xie, Robert, & Terashima, 1994; Eglinton et al., 2000; Terashima, Hafrén, Westermark, & VanderHart, 2002; Terashima et al., 2009). However, the microscopic distribution of endogenous coniferin in living plants has not been directly visualized because the cellular distribution of such a water‐soluble, small molecule could be easily altered by typical sample preparation procedures for microscopic observations, including cutting, solvent‐exchanging, resin‐embedding, and drying steps.

Recently, the authors successfully visualized the cellular distribution of endogenous coniferin in freeze‐fixed stems of *Ginkgo biloba* L. using cryo‐time‐of‐flight secondary ion mass spectrometry (cryo‐TOF‐SIMS). Each living plant sample was quickly frozen, and the frozen, hydrated transverse surface was directly analyzed by mass spectrometry and electron microscopy techniques. The authors concluded that coniferin is stored in differentiating xylem vacuoles and used as a lignin precursor (Aoki et al., 2016; Aoki, Matsushita, & Fukushima, 2018).

Another common structural unit in angiosperm lignin is the syringyl unit, which is derived from sinapyl alcohol. It is currently unknown whether sinapyl alcohol glucoside, syringin, is used as a lignin precursor. Terazawa et al. (1984) reported that there are several plant species that store syringin in their living tissues (Terazawa et al., 1984). For syringin storage in such plants, for example, *Magnolia kobus* DC. and *Syringa vulgaris* L. (lilac), syringin is reported to be mainly stored in bark, and the syringin amount is large in autumn and winter (Freudenberg & Heimberger, 1951; Kurkin, Zapesochnaya, Grinenko, & Zolotarev, 1989; Kurkin, Grinenko, Zapesochnaya, Dubichev, & Vorontsov, 1992; Fukushima et al., 1996). Previously, the role of syringin as a phenolic glucoside compound stored in the phloem region was discussed in connection with its defensive characteristics (Cis, Nowak, & Kisiel, 2006; Cipollini et al., 2011). Similar compounds such as stilbene glucosides were also reported as defensive compounds, and their distribution in the phloem region was studied in detail (Jyske et al., 2016).

However, the correlation between syringin and lignification mechanisms has not been extensively studied. Experiments using isotope‐labeled syringin with lilac suggest that the axial elements in the differentiating xylem region can assimilate the sinapyl alcohol unit of syringin into lignin (Fukushima & Terashima, 1990). However, the microscopic distribution of endogenous syringin in the differentiating xylem region of living lilac stems has not been revealed, and the role of syringin in lignification mechanisms has not yet been elucidated.

In this study, we sampled lilac plants in the growing period and examined the distribution of endogenous syringin in the transverse and radial surfaces of freeze‐fixed lilac stems by cryo‐TOF‐SIMS measurements and cryo‐scanning electron microscopy (cryo‐SEM) observations. The amount and rough distribution of syringin were confirmed by high‐performance liquid chromatography (HPLC) measurements of serial tangential sections of lilac stems. The lignification stages of wood fibers were estimated by visible, polarized, and UV‐light microscopy observations. The microscopic distribution of syringin in the differentiating xylem region was discussed with respect to the lignification stages.

## 2 EXPERIMENTAL PROCEDURES

### 2.1 Plant materials and reagents

A sample disk (thickness of 10 mm) was obtained from *S. vulgaris* grown in the Botanic Garden of Hokkaido University and cut into small blocks (circular sector with a radius of 5 mm and central angle of π/16 for chromatography, π/4 for TOF‐SIMS) containing bark, cambial zone, and xylem on June 14, 2016 in Hokkaido, Japan. The blocks were quick‐frozen with liquid Freon^®^ 22 (DuPont) at −160°C and stored at −80°C. The sample preparation procedures for cryo‐TOF‐SIMS, chromatography, and microscopic observations are schematically illustrated in Figure S6. Coniferin, syringin, coniferyl alcohol, and sinapyl alcohol were synthesized by the same method described by Terashima, Ralph, and Landucci (1996).

### 2.2 Chromatography measurements

A frozen block was cut into serial tangential sections that were 100 μm thick from the bark to the xylem. Each section was extracted using 1 ml of water for 30 min at 100°C and 2.5 hr at 25°C. The obtained extracts were analyzed by HPLC to quantify the monolignols and their glucosides. HPLC measurements were performed using a Shimadzu SPD‐1An apparatus (Shimadzu Corp). The measurement conditions were as follows: column: TSK‐gel ODS‐100S (4.6 mm I.D. × 250 mm, Tosoh Corp); flow rate: 1.0 ml/min; temperature: 40°C; detection wavelength, 260 nm; mobile phase consisting of (A) ultrapure water and (B) MeOH: acetonitrile = 6:1. The linear gradient elution was as follows: B 10% 10 min, linear B 10%–20% 10 min, linear B 20%–45% 60 min, linear B 45%–10% 5 min, and B 10% 5 min, for a total of 90 min. The measurements were performed in triplicate using three blocks from the same disk to evaluate the average amount and standard deviation of the target chemicals.

### 2.3 Cryo‐TOF‐SIMS/SEM analyses

The details of the manufactured cryo‐TOF‐SIMS/SEM system were previously described (Kuroda et al., 2013; Masumi et al., 2014). The frozen sample block (central angle π/16) was fixed in a Cu sample holder by ice embedding. After cutting the block to form a clean and flat surface in a glove box under a dry N_2_ atmosphere (<−10°C), the block was transferred to the cryo‐TOF‐SIMS by a cryo‐vacuum shuttle. Positive ion images were obtained by cryo‐TOF‐SIMS (TRIFT III spectrometer, ULVAC‐PHI Inc.). The measurement conditions were as follows: primary ion, 22 keV Au_1_^+^ at a current 5–7 nA; raster size 400 × 400 μm or 200 × 200 μm (256 × 256 pixels); pulse width, 1.8 ns (bunched for spectrum) or 13.0 ns (non‐bunched for image); mass range, *m*/*z* 0.5–1,850; spot size, 1.0 μm in non‐bunched mode; temperature, −120 to −130°C; and a low‐energy pulsed electron gun (30.0 eV) was used for surface charge compensation. The aqueous solutions of standard chemicals were frozen and measured by cryo‐TOF‐SIMS in the same procedure in bunched mode.

After the cryo‐TOF‐SIMS measurements, the plant sample blocks were transferred to the cryo‐SEM by a cryo‐vacuum shuttle, and the same surfaces were observed after a freeze‐etching treatment to enhance the image contrast in the cryo‐SEM images because the sample surfaces maintained their frozen, hydrated state after the cryo‐TOF‐SIMS measurements. The observation conditions were as follows: acceleration voltage, 1.5 kV; temperature, −120°C for observation and −90°C for freeze‐etching; and working distance, 10 mm.

All the TOF‐SIMS data were obtained as “RAW” data files, and a full mass spectrum was recorded at every 256 × 256 pixel points. The obtained images were connected using WinCadence 5.1.2.8 (ULVAC‐PHI Inc) and MATLAB R2014a (The MathWorks Inc) with PLS Toolbox 7.5.2 (Eigenvector Research Inc) without any ion count normalization. The color scale for the obtained united image was changed by ImageJ software (The National Institutes of Health) (Rasband, 19972014). The continuous SEM images were prepared using Photoshop CS5 Extended (Adobe Systems Inc).

## 3 RESULTS AND DISCUSSION

### 3.1 Radial quantitative distribution of monolignol glucosides by HPLC

**Figure 1:**
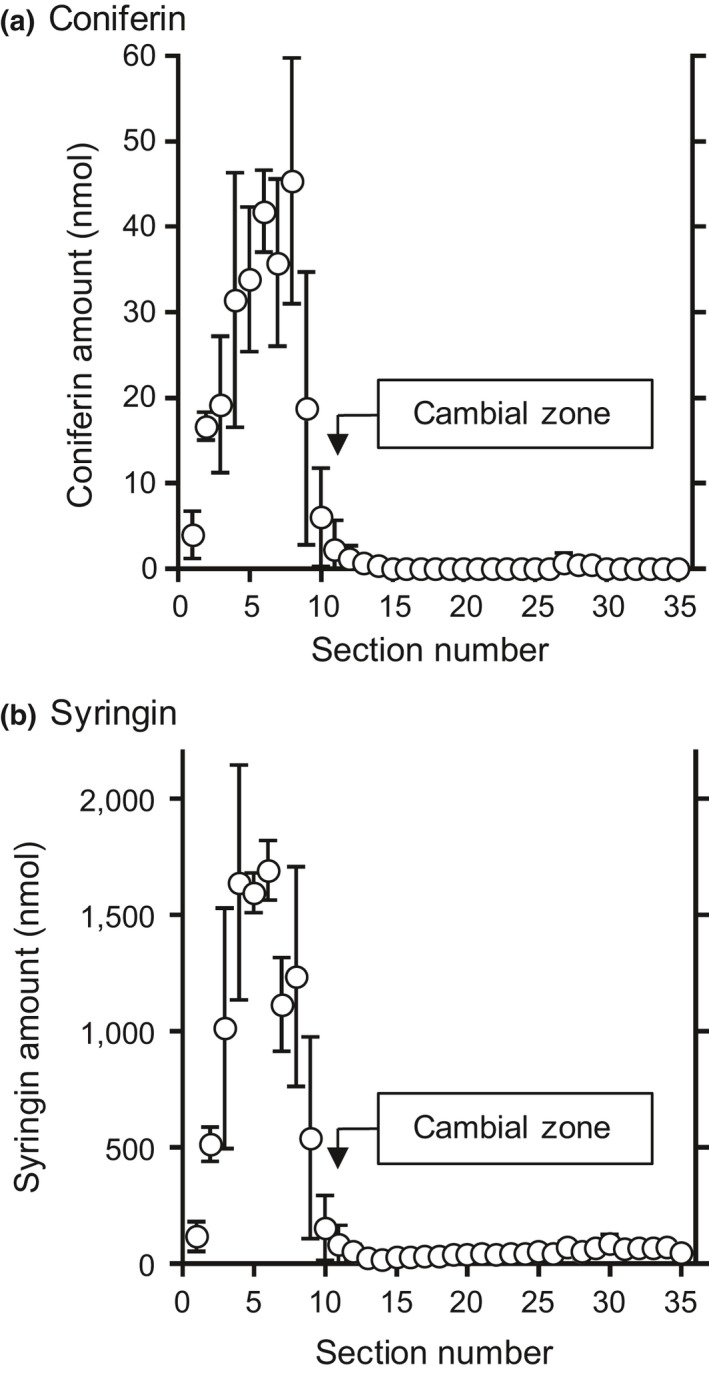
The radial distributions of (a) coniferin and (b) syringin quantified by HPLC using the serial tangential sections. The position of the cambial zone corresponding to section No. 11 was determined by the dry weight variation in the sections, as shown in Figure S1. The means and standard deviations for each section were obtained from three sets of measurements (*n* = 3) using different sample blocks cut from the same disk

To evaluate the actual amounts and radial distributions of monolignol glucosides, coniferin, and syringin in lilac, a freeze‐fixed block sample was cut into serial tangential sections that were 100 μm thick, and the sections were extracted using hot water. The coniferin and syringin amounts in each section were quantified using HPLC, and the results are summarized in Figure 1. The amounts in the section containing the cambial zone (No. 11) were determined using the dry weight variation in the sections, as shown in Figure S1.

Both coniferin and syringin were mainly distributed in the bark region (No. 1–10) and were detected in small amounts in the cambial zone and the differentiating xylem region (No. ca. 11–16). A small amount of syringin was also found in the lignified xylem region (No. ca. 17–35), but coniferin was barely detected in the region. Overall, syringin was much more abundant than coniferin. These results indicate that syringin is more abundant than coniferin and is mainly stored in bark, which is consistent with previous studies (Terazawa et al., 1984; Kurkin et al., 1989, 1992). The free monolignols corresponding to the aglycon units of the monolignol glucosides, coniferyl alcohol, and sinapyl alcohol were present in trace amounts in lilac (Table S1); however, it was difficult to quantify them in tangential sections.

### 3.2 Cryo‐TOF‐SIMS spectra of standard chemicals and freeze‐fixed lilac stems

**Figure 2:**
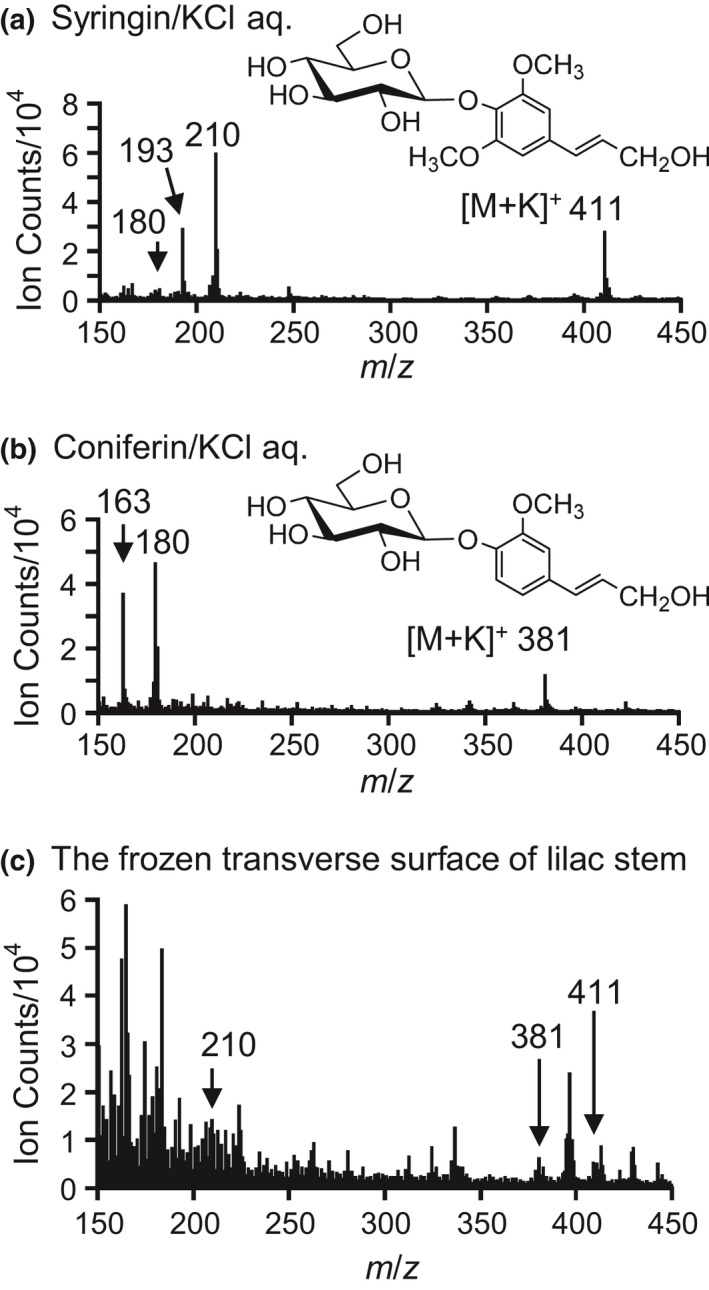
Cryo‐TOF‐SIMS spectra and chemical structures of (a) syringin and (b) coniferin; (c) cryo‐TOF‐SIMS spectrum obtained from frozen, hydrated transverse surface of a lilac stem in the region containing the cambial zone and the differentiating xylem. Syringin and coniferin were dissolved at a 10 mM concentration in a 10 mM KCl aqueous solution and frozen for measurement

In TOF‐SIMS measurements, the chemical environment surrounding target chemicals can alter the resultant mass spectrum, which is called a matrix effect. In particular, inorganic elements strongly affect the spectrum. To imitate the chemical environment in plants, KCl was added to the standard aqueous solutions because K is the most abundant inorganic element in plants (Kirkby, 2012; Wang, Zheng, Shen, & Guo, 2013). As previously reported (Aoki et al., 2016; Okumura, Aoki, Matsushita, Yoshida, & Fukushima, 2017) and shown in Figure 2a,b, the characteristic secondary ions of syringin and coniferin are the potassium adduct molecular ions [M+K]^+^ (*m*/*z* 411 ion for syringin and *m*/*z* 381 ion for coniferin), aglycon fragments (*m*/*z* 210 ion for syringin and *m*/*z* 180 ion for coniferin), and dehydrated aglycon fragments [aglycon‐OH]^+^ (*m*/*z* 193 ion for syringin and *m*/*z* 163 ion for coniferin).

If syringin and coniferin can be independently visualized, we can examine their differences in freeze‐fixed plant stems. Unfortunately, the potassium adduct molecular ions of coniferin can overlap with potassium adduct disaccharide ions (C_12_H_22_O_11_K) at *m*/*z* 381 (Aoki et al., 2016). The distribution of saccharides in lilac stems was evaluated by ion chromatography, and the results are summarized in Figure S2. Sucrose was more abundant than coniferin, and its distribution overlapped with that of coniferin. Furthermore, the *m*/*z* 180 ion can also be produced by syringin, probably as a de‐methoxylated aglycon fragment (Figure 2a). Although syringin produced fewer *m*/*z* 180 ions, the actual molar abundance of syringin was a few ten‐fold larger than that of coniferin, and their distributions were similar, as confirmed by HPLC measurements (Figure 1). Based on this information, we concluded that we could not visualize coniferin in freeze‐fixed lilac stems using cryo‐TOF‐SIMS in this study.

An example of a cryo‐TOF‐SIMS spectrum obtained for the differentiating xylem region of the frozen, hydrated transverse surface of a lilac stem is shown in Figure 2c. Secondary ions were observed at *m*/*z* 411, 381, and 210. If no additional overlapping compounds are present, the *m*/*z* 210 ion should be better for identification because it has a higher intensity than the *m*/*z* 411 ion. However, we know that plants often contain another compound that produces a secondary ion at *m*/*z* 210, as suggested in a previous study using *M. kobus* (Okumura et al., 2017). Fortunately, the lilac sample contained abundant syringin, and we could detect the *m*/*z* 411 ion. However, if syringin is present in a cell without potassium, we cannot visualize syringin using the *m*/*z* 411 [M+K]^+^ ion. Considering these points, we selected both *m*/*z* 210 and 411 ions for syringin visualization.

### 3.3 Distribution of syringin in a freeze‐fixed lilac stem

**Figure 3:**
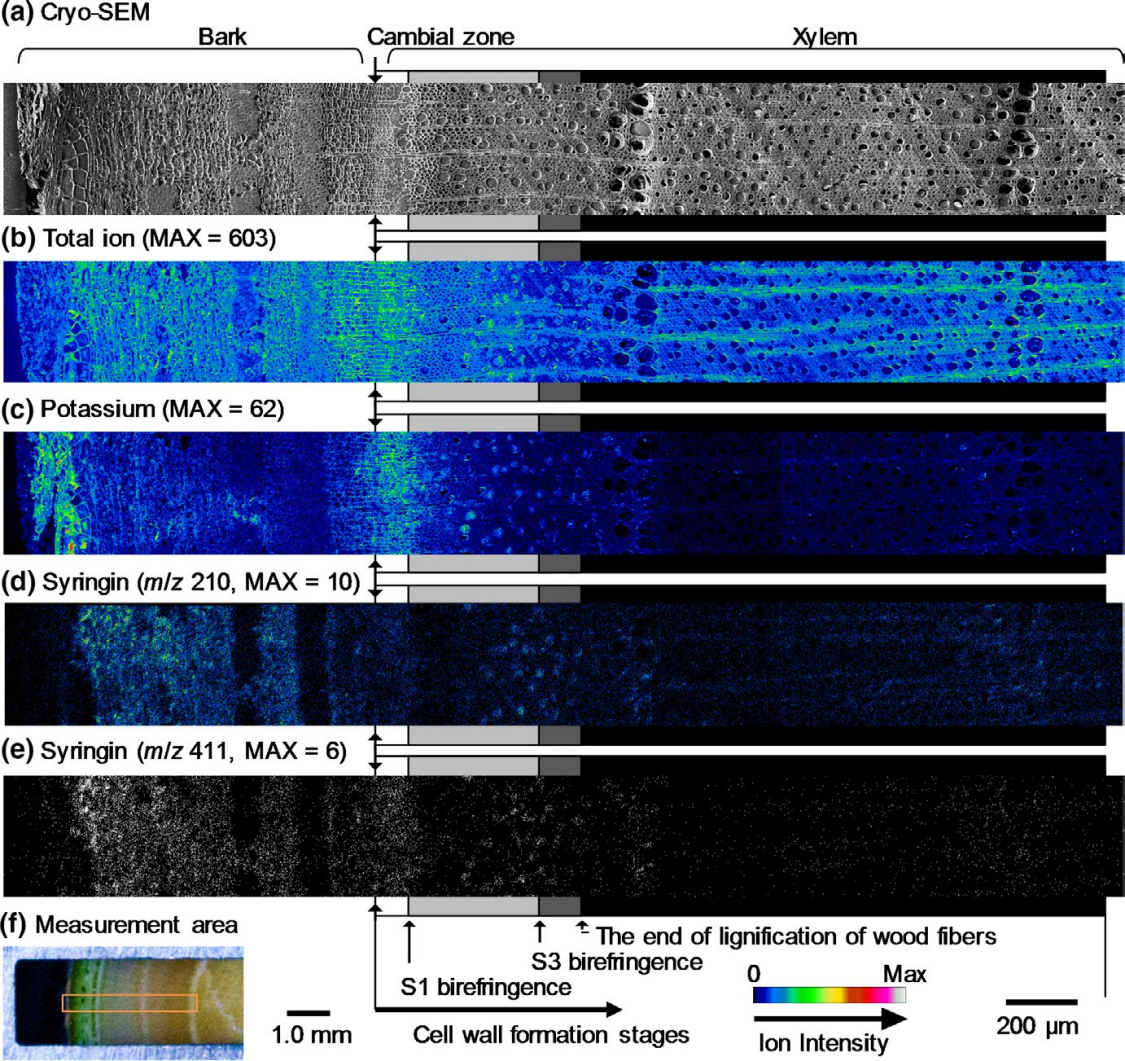
Transverse surface images of a freeze‐fixed lilac stem by cryo‐TOF‐SIMS/SEM. (a) Cryo‐SEM image taken after cryo‐TOF‐SIMS measurements and an appropriate freeze‐etching step. Cryo‐TOF‐SIMS‐positive ion images of (b) total ions, (c) K^+^ at *m*/*z* 38.96, (d) syringin at *m*/*z* 210, and (e) syringin at *m*/*z* 411 (binary image). (f) Optical microscopy image of the transverse surface of a freeze‐fixed lilac stem in the cryo‐TOF‐SIMS/SEM sample holder showing the measurement area. Grayscale tetragons on both sides of the images of (a–d) suggest the cell wall formation stages of wood fibers. Scale bars are 100 μm for (a–e) and 1.0 mm for (f)

The resultant images obtained by cryo‐TOF‐SIMS/SEM measurements of a freeze‐fixed lilac stem are displayed in Figure 3. After the cryo‐TOF‐SIMS measurements, the frozen sample was transferred for cryo‐SEM analysis, and the same surface was observed after appropriate freeze‐etching. In Figure 3, the images obtained using cryo‐SEM and the cryo‐TOF‐SIMS total ions, potassium ions, and syringin ions obtained for the same region are displayed. The measurement area of the sample surface containing bark, the cambial zone, and xylem is shown in Figure 3f. The cell wall formation stages of wood fibers were determined by microscopic observations (Figures S3 and S4) and are annotated using grayscale tetragons on both sides of the images.

Potassium (Figure 3c) was distributed in almost all of the region at the measurement surface. The strongest detection was in the outer bark and the early differentiating xylem region in which primary walls/S1 layers of wood fibers were forming. After S1 formation (S1 birefringence appearance), the potassium amount in the axial elements slightly decreased. In the later differentiating xylem region in which the S2 and S3 layers of wood fibers were forming, more potassium was found in the vessels than in the other cells. After lignification, potassium was seldom detected in the vessels. Based on this result, the xylem vessel sap content should vary with the differentiating and lignifying stages of the surrounding wood fibers. A similar situation was previously reported for *M. kobus* using cryo‐TOF‐SIMS/SEM measurements (Okumura et al., 2017).

The syringin visualization based on *m*/*z* 210 (Figure 3d) and 411 (Figure 3e) ions indicated nearly the same distribution. Syringin (Figure 3d,e) was detected in a wide area of the bark region except for the phloem fibers and sclereids and outer bark. Tissue classification of the bark region was performed by microscopy observations (Figure S5). The abundant presence of syringin in the bark region agreed with the HPLC results (Figure 1) and previous reports (Terazawa et al., 1984; Kurkin et al., 1989, 1992). Therefore, we concluded that both the *m*/*z* 210 and 411 ions represent the syringin distribution in freeze‐fixed lilac stems.

In the differentiating xylem region, the syringin distribution varied with the cell wall formation stages. From the cambial zone to the early differentiating region, in which primary walls and/or S1 layers of wood fibers are still forming, syringin was evenly distributed irrespective of the cell type. After S1 layer formation, syringin detection decreased. However, in the latter part of the S2 and S3 layer forming period (indicated by light gray tetragons), syringin was abundant in vessels. After wood fiber lignification, some syringin was detected in rays and some vessels in the initial part of the annual rings. Syringin was also detected in the previous annual ring region by HPLC measurements (Figure 1, No. ca. 17–35).

**Figure 4:**
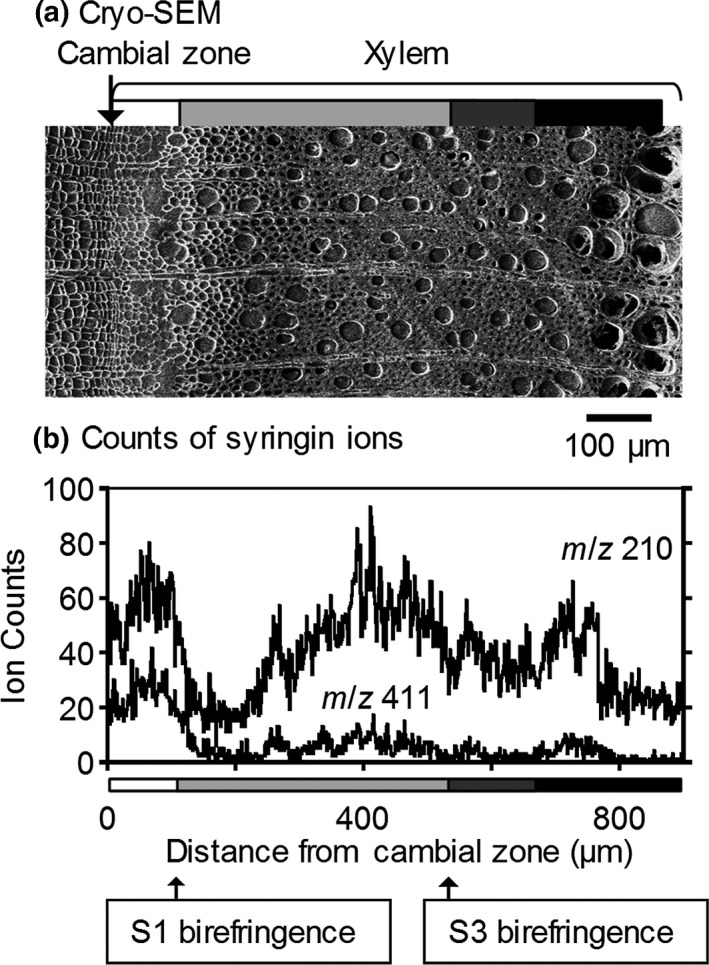
(a) Cryo‐SEM transverse surface image showing the area from the cambial zone to the end of cell wall formation. (b) Radial distribution of *m*/*z* 210 and *m*/*z* 411 ions in the same region of (a). Grayscale tetragons in the lower side of (b) suggest the cell wall formation stages of wood fibers. The scale bar is 100 μm

**Figure 5:**
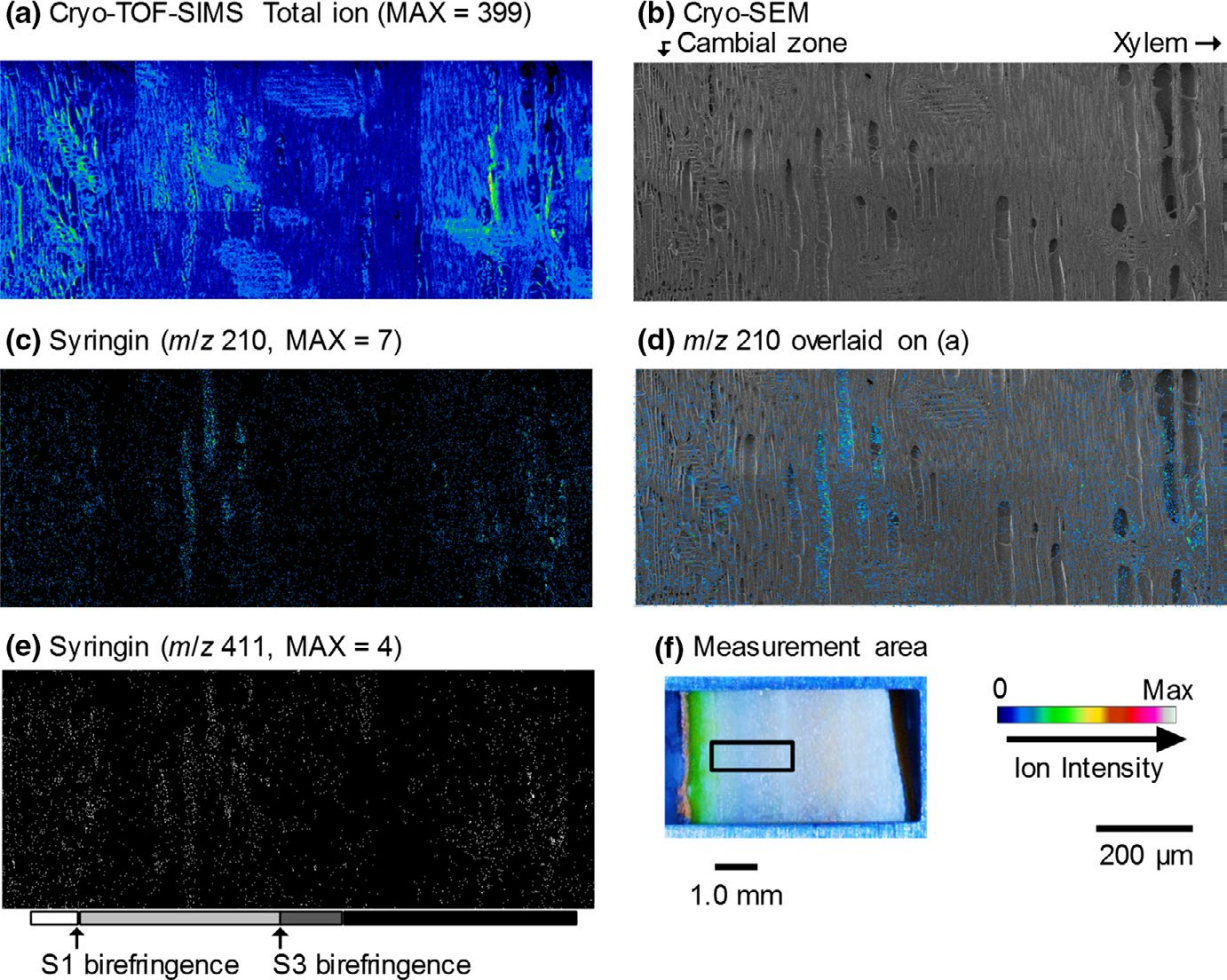
Radial surface images of the freeze‐fixed lilac stem by cryo‐TOF‐SIMS/SEM. (a) Cryo‐TOF‐SIMS total ion image. (b) Cryo‐SEM image taken after the cryo‐TOF‐SIMS measurement and freeze‐etching step. Cryo‐TOF‐SIMS‐positive ion images of (c) syringin at *m*/*z* 210, and (e) syringin at *m*/*z* 411 (binary image). (b) Cryo‐SEM image with *m*/*z* 210 overlay. (f) Optical microscopy image of the radial surface of a freeze‐fixed lilac stem showing the measurement area. Scale bars are 200 μm for (a, b, c, d, e) and 1.0 mm for (f). Grayscale tetragons in the lower side of (e) suggest the cell wall formation stages of wood fibers, which were estimated by the results obtained for the transverse surface, as shown in Figures 3 and 4

To estimate the syringin distribution in the differentiating xylem region in detail, the radial variations in the *m*/*z* 210 and 411 ion counts are shown in Figure 4. The syringin ion counts increased from the cambial zone and decreased after the deposition of the S1 layer of wood fibers. As indicated in Figure 3, the ion count increase was derived from that in the vessels in the later differentiating regions in which the S2 and S3 layers were deposited. To confirm the distribution of syringin, the radial surface of a freeze‐fixed lilac stem was measured by cryo‐TOF‐SIMS/SEM. The resultant images are displayed in Figure 5. The *m*/*z* 210 and 411 ions clearly indicated syringin storage in the early differentiating xylem region and vessels.

### 3.4 Syringin distribution and lignification stages

In a previous study administering coniferin and syringin to lilac, isotope‐labeled monolignol units were incorporated into the lignin of the axial elements (Fukushima & Terashima, 1990). This result suggests that the axial elements in the differentiating xylem region can assimilate syringin into lignin. In this study, endogenous syringin was stored in the axial elements in the early lignification stages, and the amount was observed to decrease after S1 layer formation.

Cryo‐TOF‐SIMS can visualize the apparent concentration of target compounds but cannot display the actual biosynthetic activity at the surface when it is frozen. If syringin is actively assimilated into lignin in the cell, the apparent concentration and cryo‐TOF‐SIMS detection of syringin should decrease. Correspondingly, the differentiating xylem cells have the ability to assimilate syringin into lignin and store syringin in the cell at the same time; the storage amount of syringin is correlated with the lignification stage. Based on these points, the authors conclude that the syringin stored in the differentiating xylem region should be used as a lignin precursor.

Syringin detection in the vessels in the latter lignification stages may indicate other syringin behavior. Generally, the lignification of vessels is complete before that of neighboring wood fibers. Therefore, the purpose of syringin storage in vessels is not likely to be lignification. Here, the authors suggest the possibility of the intercellular transportation of syringin within wood fibers, vessels, and rays. Thus, young and biosynthetically active cells produce cell wall precursors and transfer them to the older cells in the inner xylem region; then, the vessels act as a precursor depository in the latter lignification stages. The relatively small lumen volume of lilac wood fibers may be one motivation for such a behavior. Intercellular transportation of lignin precursors has been discussed from the perspective of postmortem lignification (Hosokawa, Suzuki, Umezawa, & Sato, 2001; Pesquet et al., 2013). The increment of the syringyl to guaiacyl ratio of the lignin structural units in the latter stage of angiosperm lignification (Terashima, Fukushima, & Takabe, 1986; Terashima & Fukushima, 1989; Fukushima & Terashima, 1990; Table S2) may be considered in discussions regarding the regulatory mechanisms of lignification in future studies.

In lilac, the majority of syringin is stored in the phloem region, and the primary purpose of syringin might not be to act as a lignin precursor. However, our present data suggest that syringin stored in the differentiating xylem region works as a lignin precursor. Additionally, the characteristic distribution of syringin suggests intercellular transportation of syringin in lilac stems.

## CONFLICT OF INTEREST

The authors declare no conflict of interest associated with the work described in this manuscript.

## AUTHORS CONTRIBUTION

DA, WO, YM, and KF designed the research. DA, WO, and YS collected the lilac stem samples. WO and YM prepared the standard chemicals. DA, WO, and TA operated cryo‐TOF‐SIMS/SEM and HPLC experiments. WO, MY, and YS conducted microscopic observations and tissue assignments. All authors discussed the results. DA and WO wrote the manuscript.
