# Supplementary material for: Microscopic distribution of syringin in freeze‐fixed Syringa vulgaris stems
Source: Plant Direct. 2019 Aug 2;3(8):e00155. doi: 10.1002/pld3.155 (PMC6676449; doi:10.1002/pld3.155)
Supplement: Supplementary file 1 [file PLD3-3-e00155-s001.pdf]

Supporting Information

Microscopic distribution of syringin in freeze-fixed *Syringa vulgaris* stems

Dan Aoki[1*], Wakaba Okumura[1], Takuya Akita[1], Yasuyuki Matsushita[1], Masato Yoshida[1], Yuzou Sano[2], and Kazuhiko Fukushima[1]

[1] Graduate School of Bioagricultural Sciences, Nagoya University, Nagoya, Aichi 464-8601, Japan
[2] Research Faculty of Agriculture, Hokkaido University, Sapporo 060-8589, Japan

* Corresponding author
E-mail address: daoki@agr.nagoya-u.ac.jp

Table S1. Quantification results of coniferyl alcohol and sinapyl alcohol.

Table S2. Yields of thioacidolysis monomer products.

Figure S1. Dry weight variation of the serial tangential sections with a 100-μm thickness used for chromatography measurements.

Figure S2. The radial distribution of sucrose, glucose, and fructose evaluated by ion chromatography measurements using serial tangential sections with a 100-μm thickness.

Figure S3. Cryo-SEM image and images of the transverse section of a resin-embedded lilac stem observed by visible light with toluidine blue staining, polarized light, and UV light.

Figure S4. Procedure for the determination of the cell wall formation stages of wood fibres by polarized optical microscopy (POM) and UV microscopy.

Figure S5. Comparative visualization of images obtained by cryo-SEM and the cryo-TOF-SIMS total ion and overlay of cryo-TOF-SIMS *m*/*z* 411 ion (red) on the cryo-SEM image illustrating the syringin distribution in the freeze-fixed stem bark of *S. vulgaris*.

Figure S6. Schematic illustration of the sample preparation.

Table S1. Quantification results of coniferyl alcohol and sinapyl alcohol.

	mol-% to syringin	mol-%S.D. to syringin
coniferin	3.32	0.82
coniferyl alcohol	0.57	0.21
sinapyl alcohol	0.19	0.09

To quantify coniferyl alcohol and sinapyl alcohol, a freeze-fixed block sample was cut into serial tangential sections that were 100-µm thick, and the sections were put into the same vial bottle and extracted using hot water. The elution times for coniferin, syringin, coniferyl alcohol, and sinapyl alcohol were 21.5 min, 24.8 min, 28.3 min, and 28.7 min, respectively. Their amount was quantified and represented as mol% values to syringin. The measurements were performed 5 times using 5 different blocks (n=5) from the different sample disks to evaluate the average amount and standard deviation of the target chemicals.

Table S2. Yields (μmol/sample-g) of thioacidolysis monomer products.

	G	G (S.D.)	S	S (S.D.)	S/G
Phloem	12.6	2.6	8.4	2.5	0.67
Differentiating xylem	77.7	29.3	36.9	4.0	0.47
Mature xylem	142.3	22.3	244.4	27.9	1.72

To evaluate syringyl (S) to guaiacyl (G) ratio of lignin structural units, thioacidolysis followed by GC-MS measurements was conducted. A sample block was cut into serial tangential sections and the sections were classified to 3 regions of phloem, differentiating xylem, and mature xylem (lignification completed) in referring to Fig. S1 and S3. The means and standard deviations for each region were obtained from three sets of measurements (n=3) using different sample blocks cut from the same disk.

Thioacidolysis and GC-MS measurement conditions are as follows. The sections were dipped in 2.5 mL of dioxane/ethanethiol/$(C_2H_5)_2OBF_3$ mixture (35/4/1, vol./vol./vol.), and 0.5 mg of docosane in dioxane was added as an internal standard. The reaction was conducted at 100 °C for 4 hours. The reaction mixture was cooled to room temperature, and then 0.4 M $NaHCO_3$ aq. was added to the mixture to stop the reaction. The mixture was adjusted to pH 2–3 with diluted hydrochloric acid aq. (HCl:H_2O=1:3, vol./vol.) and then extracted by CH_2Cl_2. The extract was dried over Na_2SO_4 and concentrated. Finally, the thioacidolysis monomeric product was dissolved in 1.5-mL CH_2Cl_2 and stored at 4 °C until use. The silylated samples were analysed by GC-MS with the following condition: apparatus, TRACE 1300GC/ITQ 900 (Thermo Fisher Scientific K.K.); column, Rxi-1 ms (30 m × 0.32 mm I.D., 0.25 μm film thickness); temperature program, 180 °C 1 min, 180–230 °C 25 min, 230–300 °C 4 min 20 s, 300 °C 5 min; column flow, 15.0 mL/min; carrier gas, He; injection temperature, 250 °C. The monomeric products were assigned and evaluated by GC according to the previous report (Roland et al. 1992).

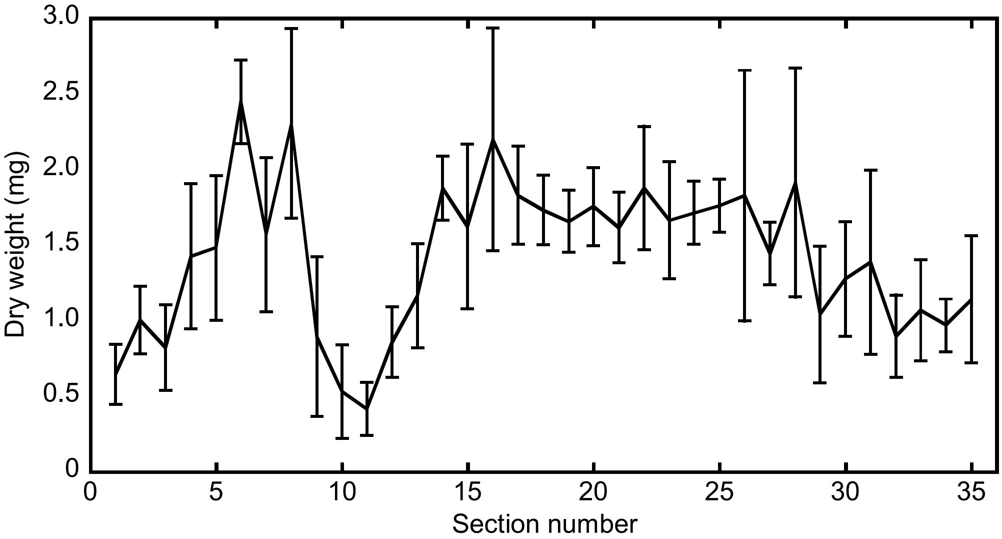

Figure S1. Dry weight variation of the serial tangential sections with a 100-μm thickness used for chromatography measurements. The means and standard deviations for each section were obtained from three sets of measurements (n=3) using the different sample blocks cut from the same disk.

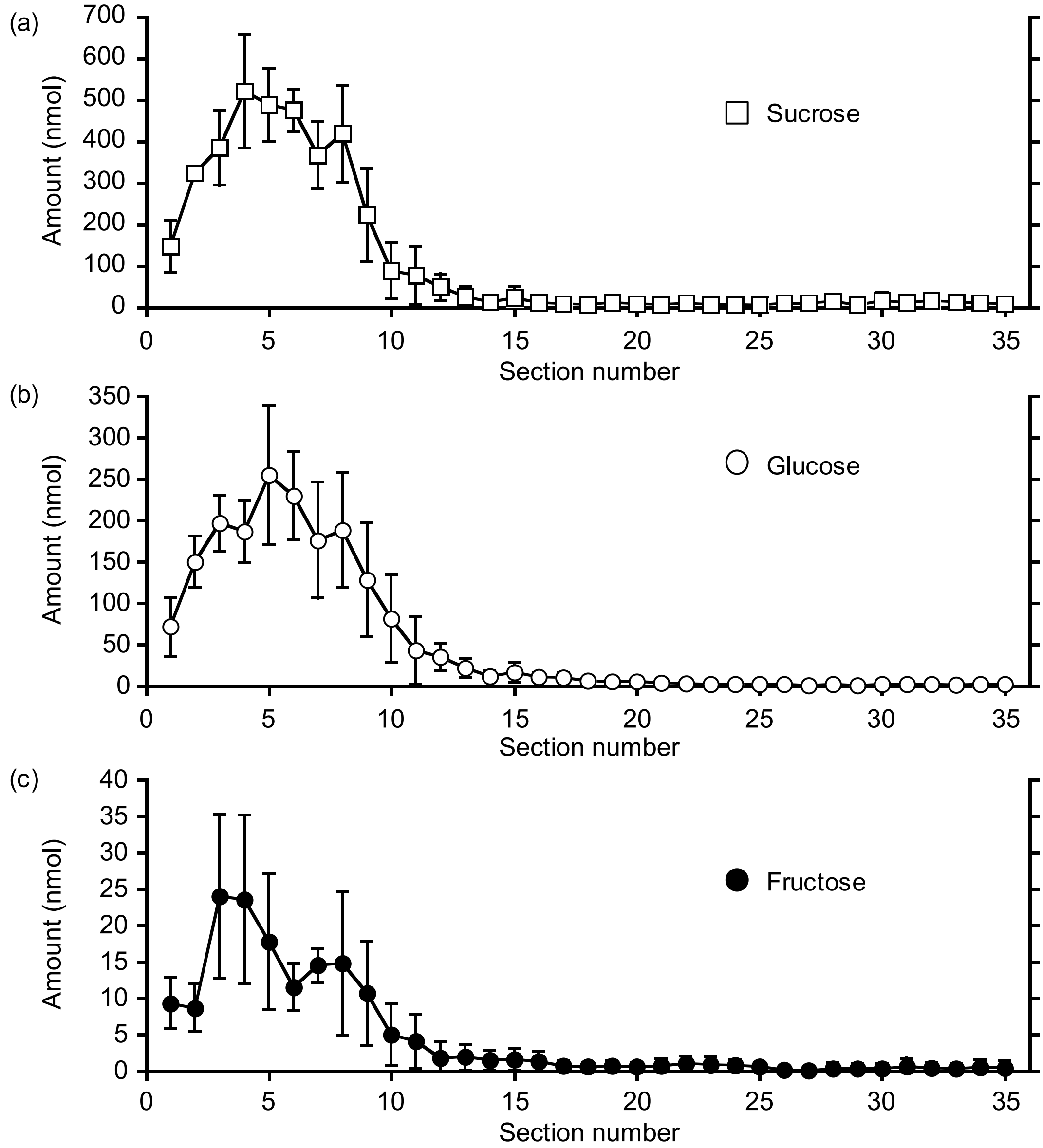

Figure S2. The radial distribution of (a) sucrose, (b) glucose, and (c) fructose evaluated by ion chromatography measurements using serial tangential sections with a 100-μm thickness. The means and standard deviations for each section were obtained from three sets of measurements (n=3) using the different sample blocks cut from the same disk. The position of cambial zone corresponding to the section number 11 was determined by the dry weight of the sections as shown in Figure S1.

Ion chromatography measurements were conducted using a DIONEX ICS-3000 apparatus. The measuring conditions were as follows: column, CarboPac PA-1 (2.0 mm I.D. × 250 mm, Dionex corp.); flow rate, 0.3 mL min^{-1}; temperature, 30 °C; eluent, H_2O (solvent A), 100 mM NaOHaq (solvent B), and aqueous solution containing 100 mM NaOH and 1.0 M CH_3COONa (solvent C) with a gradient of B 50% C 0 % 50 min, C 100 % 10 min, B 100 % 10 min, B 50 % C 0% 15 min.

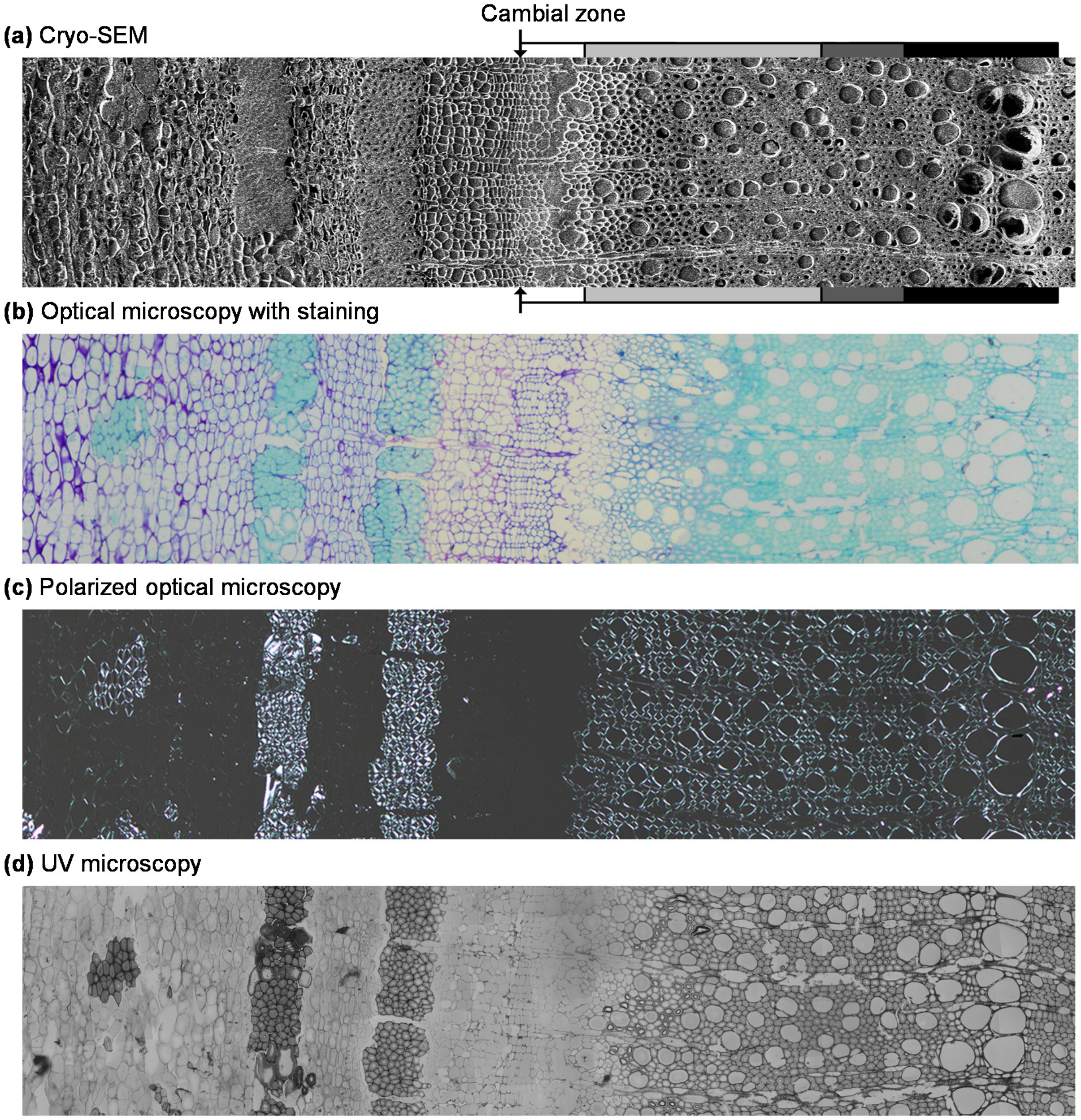

Figure S3. Cryo-SEM image and images of the transverse section of a resin-embedded lilac stem observed by visible light with toluidine blue staining, polarized light, and UV light.

To observe nearly the same surface as was measured by cryo-TOF-SIMS/SEM, the same frozen block of *S. vulgaris*, after the cryo-TOF-SIMS/SEM analysis, was immersed into glutaraldehyde-acetone solution at −80 °C for 3 days, and then stored at −30 °C for 1 day, at 4 °C for 1 day, and at room temperature. The sample block was embedded in an epoxy resin, and cut to thin sections of 1 or 2-µm thickness by a microtome (RM2155, Leica). The section of 2-µm thickness was stained by toluidine-blue and observed by optical microscope (BX50, Olympus Corp.). The sections of 1-µm were submitted to polarized optical microscopy (BX50, Olympus Corp.) and UV microspectrophotometer (MPM800; Carl Zeiss) observations. The continuous images were prepared using Photoshop CS5 Extended (Adobe Systems Inc.).

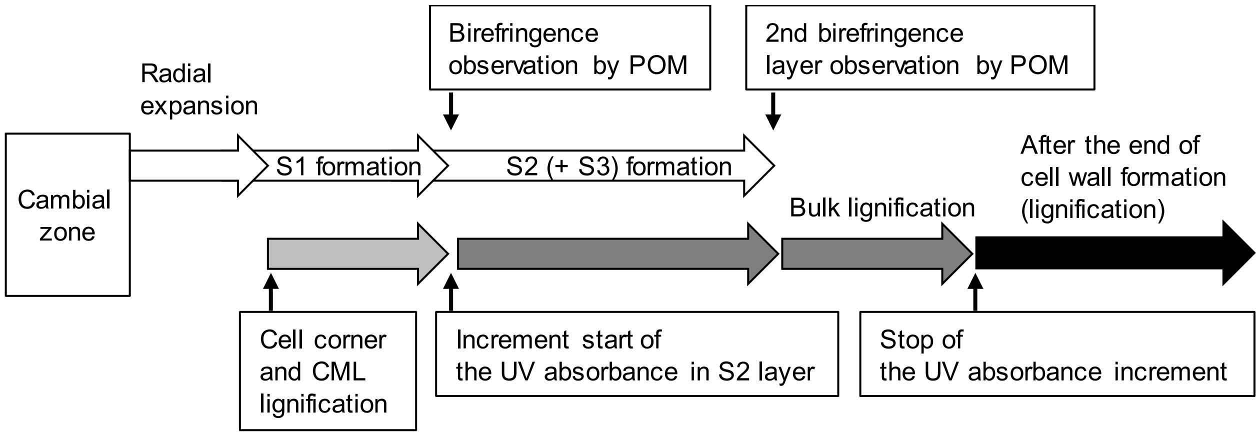

Figure S4. Procedure for the determination of the cell wall formation stages of wood fibres by polarized optical microscopy (POM) and UV microscopy.

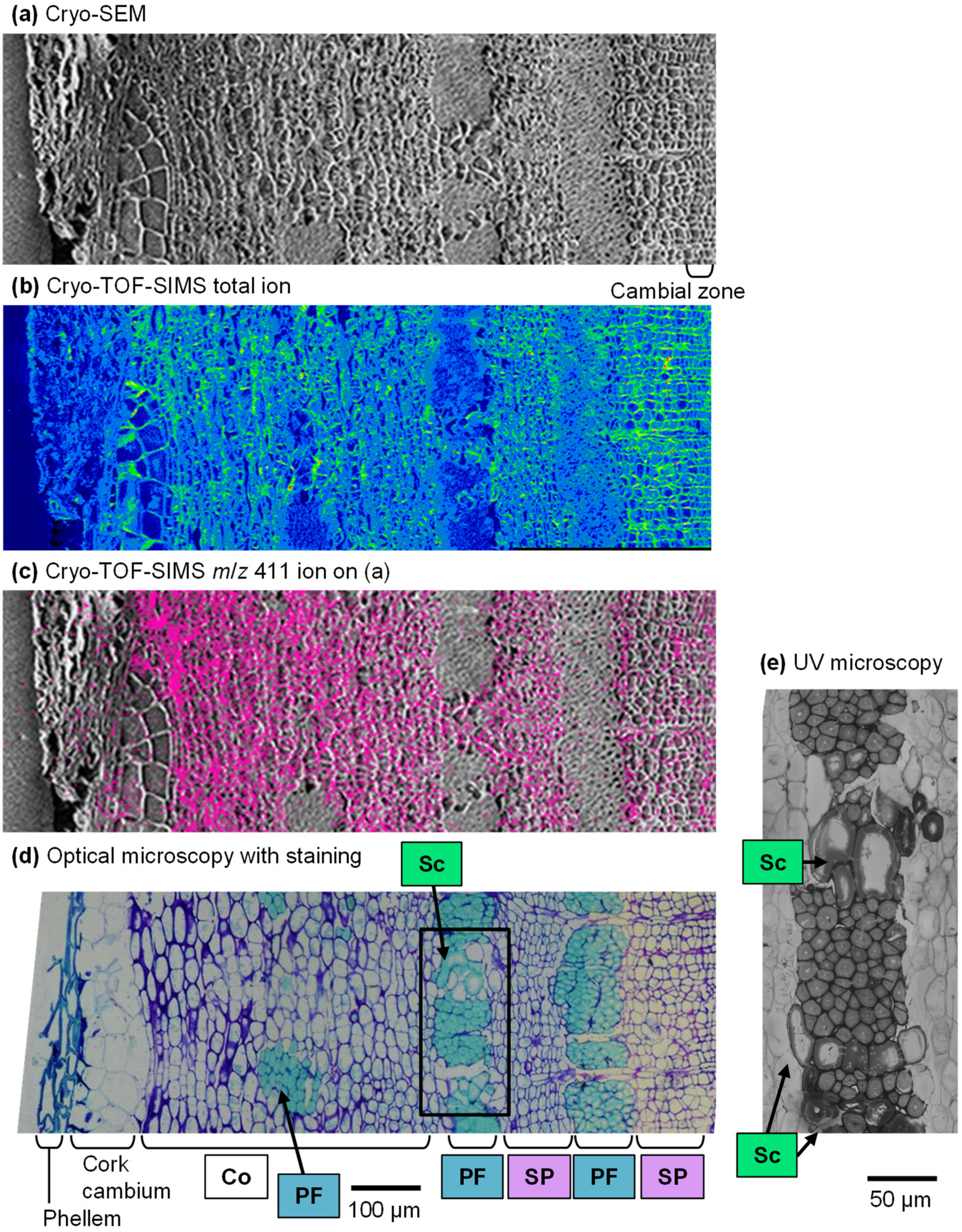

Figure S5. Comparative visualization of images obtained by (a) cryo-SEM and the (b) cryo-TOF-SIMS total ion and (c) overlay of cryo-TOF-SIMS *m*/*z* 411 ion (red) on the cryo-SEM image illustrating the syringin distribution in the freeze-fixed stem bark of *S. vulgaris*.

Nearly the same region was observed by (d) optical microscopy with toluidine blue staining and (e) UV microscopy (enlarged view of the entangled region in (d)). The letters in (d) and (e) show the positions of (Co) cortex, (PF) phloem fibre, (Sc) sclereid, and (SP) secondary phloem. Scale bars are 100 µm for (a–d) and 50 µm for (e). The overlay image was prepared using Photoshop CS5 Extended (Adobe Systems Inc.).

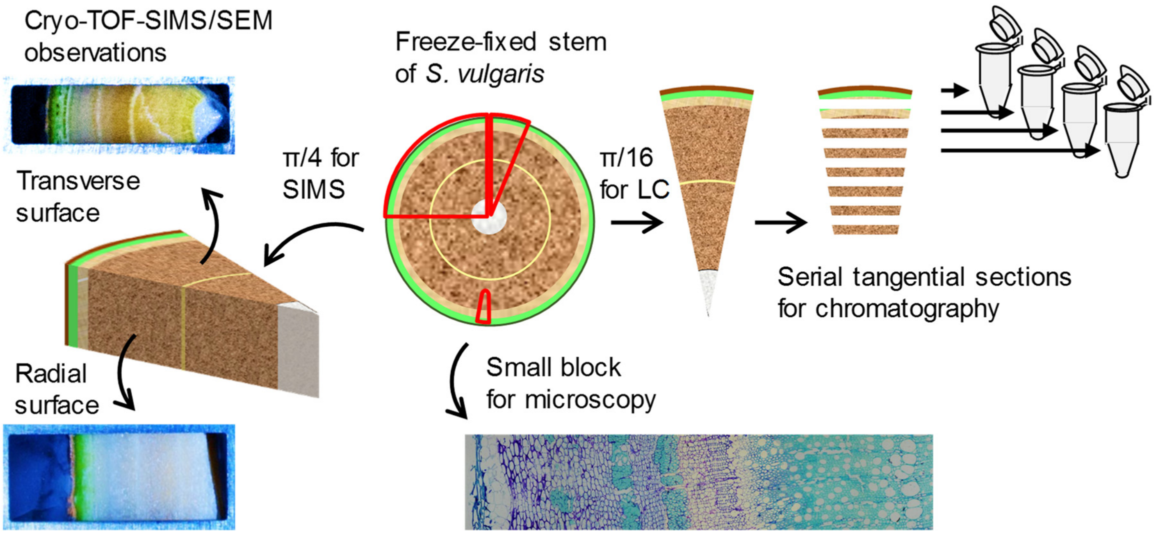

Figure S6. Schematic illustration of the sample preparation for cryo-TOF-SIMS/SEM, chromatography, and microscopic observations of freeze-fixed stem of *S. vulgaris*.

Reference

Roland, C., Monties, B., & Lapierre, C. (1992). Thioacidolysis. In S. Y. Lin, & C. W. Dence (Eds.), *Methods in lignin chemistry* (pp. 334–349). Berlin, Germany: Springer-Verlag.
