# Supplementary material for: Microscopic distribution of syringin in freeze‐fixed Syringa vulgaris stems
Source: Plant Direct. 2019 Aug 2;3(8):e00155. doi: 10.1002/pld3.155 (PMC6676449; doi:10.1002/pld3.155)
Supplement: Supplementary file 2 [file PLD3-3-e00155-s002.pdf]

Authors' responses to Editor,

> Thank you for submitting the revised version of your manuscript to Plant Direct. All the concerns raised by the three reviewers have been properly addressed, except for one, which I think is important. One of the major concerns from the original Reviewer #1 (from TPJ) is that the manuscript does not provide biochemical evidence to unequivocally demonstrate that syringin is a lignin precursor. In fact, in your response to reviewers letter, you agree and suggest that the proper phrase to use is "the data presented ... might (may?) suggest that syringin works as a lignin precursor". Yet in the main manuscript, and in at least in 3 occasions according to my own reading, including the Abstract, Significant statement and the last paragraph of Results and discussion you state that "syringin acts as a lignin precursor'

> I would ask you then to revise the entire manuscript and rephrase any number of sentences needed to denote that the role of syringin as a lignin precursor is a suggestion. After this minor modification, I will be happy to recommend your manuscript to be accepted in Plant Direct. I would also suggest that you ask a native English speaker to comment on the manuscript as I think that the word choice and phrasing could be improved as well.

Thank you for your positive decision and constructive comments for our revised manuscript.
We agree with the suggested revision.
As the editor suggested, we revised the following 4 occasions as follows.

Abstract:

> Syringin storage in the lignifying axial elements suggests that syringin acts as a lignin precursor.

Based on this, our present data showing the syringin storage in the differentiating xylem region and its variation depending the lignification stages may suggest that syringin works as a lignin precursor.

Significance statement

> The role of syringin as a lignin precursor in the differentiating xylem region was revealed, and intercellular transportation of syringin was indicated.

The syringin storage in the differentiating xylem region and its variation depending the lignification stages may suggest the role of syringin as a lignin precursor.

The 2nd paragraph of "Syringin distribution and lignification stages"

> Based on these points, the authors conclude that the syringin stored in the differentiating xylem region is used as a lignin precursor.

Based on these points, the authors conclude that the syringin stored in the differentiating xylem region should be used as a lignin precursor.

The last paragraph of the Results and discussion

> However, it is possible to conclude that one of the roles of syringin is a lignin precursor.

However, our present data may suggest that syringin stored in the differentiating xylem region works as a lignin precursor.

For English, we submitted our latest manuscript to Wiley editing service to request the further improvement. We attached the confirmation PDF. Now we believe that the content is adequately corrected.
Certificate verification key: 5F3E-8F53-AE6E-9FFE-3CAP

Authors' responses to reviewers

----------Plant Direct Reviewer comments:
Reviewer #1:

> The manuscript titled "Microscopic distribution of syringin in freeze-fixed Syringa vulgaris stems" by Aoki et al. describe the study of syringin distribution in stem of lilac using cryo time-of-flight secondary ion mass spectrometry and scanning electron microscopy. The results show that Syringin was mainly found in the phloem region and was evenly distributed from the cambial zone to the early differentiating stage region and selectively distributed in vessels in the later differentiating stage region. Visualizing the cellular distribution of endogenous monolignols and their derivatives in living xylem cells remains extremely challenge. The experiments and analysis were well designed and performed. This manuscript provides some new information about the distribution and function of syringin in xylem lignification. The conclusions were supported by the experimental results. I believe it is suitable to publish it on Plant Direct. A few minor changes are list below:

Thank you for your positive and constructive comments.
We have revised several points that the reviewer has pointed out as follows:

> 1. Figure 1, I wonder if it is better to make separated figures for coniferin and syringin.

Thank you for the comment. We separated coniferin and syringin in Fig. 1.

> 2. In Figure 2, it is not clear from which location the SIMS spectrum was obtained.

Thank you for pointing this out. We revised the description pertaining to where the spectrum was obtained in the Fig. 2 legend as follows:
"(c) cryo-TOF-SIMS spectrum obtained from frozen, hydrated transverse surface of a lilac stem in the region containing the cambial zone and the differentiating xylem"

> 3. In Figure 3, (a) Cryo-SEM Bark should be Cryo-SEM

Thank you for the comment, we have revised this mistake.

> 4. In section "Syringin distribution and lignification stages", "Therefore, if syringin is actively...", remove "therefore'. "However, syringin detection in the vessels.....", and "This idea means...", I would suggest remove "However" and "idea". Also, it would be good to add some references on lignification of older cells of xylems.

Thank you for your comments. We removed “therefore”, “however”, and “idea”.
Furthermore, we added a sentence with references that suggest intercellular transportation of lignin precursors: “Intercellular transportation of lignin precursors has been discussed from the perspective of post-mortem lignification (Hosokawa et al., 2001; Pesquet et al., 2013).”

__________Previous Reviewer Comments

Reviewer: 1

Reviewer Report for the Authors
> Monolignols are lignin precursors. Monolignol glucosides such as syringin and coniferin are found in plants. In this study, the authors visualize the distribution of syringin in stems of lilac (Syringa vulgaris) and show that syringin is mainly found in the bark, phloem and differentiating xylem.
Although syringin is found in most plants, its cellular distribution and its function remains unknown. This study uses cryo-TOF-SIMS/SEM and HPLC to examine the localization of syringin in vivo and the data presented in this study advances our understanding of the cellular distribution of syringin.
> However, the authors make strong conclusions without providing direct evidence. As stated in line 58-60 of page 4, it is still unknown if syringin is a precursor of lignin or not, but the authors conclude that syringin is a lignin precursor because it is found in the differentiating xylem.
> Although the differentiating xylem is where lignification occurs, syringin is predominantly found in bark and phloem as well (Fig 3, S5). Indeed, the signal of syringin in the bark and phloem seems to be stronger than that in the xylem (Fig 3). Although the localization of syringin does not necessarily conflict with the initial hypothesis that syringin can be used for lignin, further evidence is required for any substantive conclusions to be made.

Thank you for the fair and reasonable comments. As the reviewer pointed out, only our data is not enough to conclude that “syringin is a lignin precursor”. Previously, experiments have shown that angiosperms of *Syringa vulgaris* and *Magnolia kobus* can assimilate syringin into lignin in the differentiating xylem region (Fukushima and Terashima 1990). Based on this, our present data showing the syringin storage in the differentiating xylem region and its variation depending the lignification stages might suggest that syringin works as a lignin precursor. The differentiating xylem cell has the ability to assimilate and store syringin at the same time and the storage amount is correlated with the lignification stage.

As the reviewer indicated, syringin distribution is not only found in the differentiating xylem region, but also in other regions such as the phloem and mature xylem. We think that syringin may have other roles as a phenolic glucoside compound stored in the phloem region. Previously, the role of syringin was interpreted as a defensive compound (Cis et al., 2006; Cipollini et al., 2011). Similar compounds such as stilbene glucosides were also reported as defensive compounds (Jyske et al. 2016). We think that our current conclusion does not conflict with these

previous studies.

In response to the reviewers' comment; we revised the following text to add supplementary information concerning previous studies to make our conclusion reasonable.

In the "Introduction":
Previously, the role of syringin as a phenolic glucoside compound stored in the phloem region was discussed in connection to its defensive characteristics (Cis et al., 2006; Cipollini et al., 2011). Similar compounds such as stilbene glucosides, were also reported as defensive compounds and their distribution in the phloem region was studied in detail (Jyske et al., 2016).

In the section "Syringin distribution and lignification stages":
"Cryo-TOF-SIMS can visualize the apparent concentration of target compounds but cannot display the actual biosynthetic activity at the surface when it is frozen. If syringin is actively assimilated into lignin in the cell, the apparent concentration and cryo-TOF-SIMS detection of syringin should decrease. Correspondingly, the differentiating xylem cells have the ability to assimilate syringin into lignin and store syringin in the cell at the same time; the storage amount of syringin is correlated with the lignification stage. Based on these points, the authors conclude that the syringin stored in the differentiating xylem region is used as a lignin precursor."

> It is necessary to discuss the data broadly including the role of syringin in the bark and phloem and where syringyl unit and guaiacyl unit of lignin are found in lilac stem. In line 22-23 page 9, it says that monolignols are quantified using HPLC but the data are not presented. It would be informative to show if syringyl lignin and syringin are co-localized.

Thank you for the comment. We added the amounts of lignin and the S/G ratio by thioacidolysis (Table S2) and the amounts of coniferyl alcohol and sinapyl alcohol quantified by HPLC (Table S1) in Supporting Information. Table S2 presents the S to G ratio of lignin structural units in the phloem, differentiating xylem, and mature xylem region. Although this data suggests the S-unit increment in the latter stage of lignification is not new (as referred in the revised text), we suggest that the characteristic syringin distribution in this study should be involved in such a mechanism.

Based on these points, the following text were revised:

In "Radial quantitative distribution of monolignol glucosides by HPLC"
"The free monolignols corresponding to the aglycon units of the monolignol glucosides, coniferyl alcohol, and sinapyl alcohol were present in trace amounts in lilac (Table S1) however, it was difficult to quantify them in tangential sections."

In "Syringin distribution and lignification stages"
"The increment of the syringyl to guaiacyl ratio of the lignin structural units in latter stage of angiosperm lignification (Terashima et al., 1986; Terashima and Fukushima, 1989; Fukushima and Terashima, 1990; Table S2)

may be considered in discussions regarding the regulatory mechanisms of lignification in future studies."

> The picture quality of Figure 5 c and d is not clear enough to agree with the statement in line 56-58 in page 19 which says " ...clearly indicated syringin storage in the early differentiating xylem region and vessels"

Thank you for the comment. We revised Fig. 5 with the overlaid image to demonstrate the syringin distribution in vessels.

> Fig 1 and Fig S2 show the amount of monolignol glucosides and sugars. But it appears that the amount per section is shown. Considering that the dry weight of each section varies in Fig S1, it is necessary to normalize them in fresh weight or dry weight.

Thank you for the comment. As the reviewer mentioned, the values were not normalized. It is difficult to use weight for normalization because the cell wall thickness is quite different within the differentiating stages. It is also difficult to obtain fresh weights from the samples due to importance of preserving the frozen state to quantify water-soluble compounds. We believe that the "per section" transition of water-soluble compounds is adequate in the discussion of this study.

> Since various section data are presented in this study, the dimension of sections (radial surface, transverse surface, tangential sections) need to be clearly stated in the figure legends to avoid any confusion. It might be helpful if the examined area is shown in the section picture or a cartoon as shown in Aoki et al 2016.

Thank you for the suggestion. We revised figure legends to clarify the sample cutting dimensions. We added Fig. S6 as a schematic illustration of the sample preparation. The following text was added in the experimental section. "The sample preparation procedure for cryo-TOF-SIMS, chromatography, and microscopic observations are schematically illustrated in Figure S6."
Besides, the sample size description and the cutting date was corrected.

> The deviation parameter or the number of biological samples are missing in Figure 1, Fig S1, Fig S2

Thank you for the comments. We revised the figure legends.

> Page 5 line 14 to 16 need reference or data.

Thank you for pointing this out. We added the quantification results of coniferyl and sinapyl alcohols in Table S1. Their amounts were quantified within a whole sample block because of their small quantity. In Table S1, their amounts were expressed as a mol-% to syringin.

> Figure S2. The visibility of bar graphs is not good. It may be better to show the data with lines instead of bars.

Thank you for the comment. We revised Fig. S2 with a line-plot and separated them to improve readability.

> Figure 2. The legend of Fig 2c is missing.
> It is not clear which part of the stem surface is analyzed in Fig 2C. The figure title indicates that it represents the transverse surface of the stem but it says that the data was obtained from the differentiating xylem region in the result section (line 7-9 in page 6). If the specific area was chosen to identify signature mass for syringin, it needs to be explained why the area is appropriate.

Thank you for this pointing. We revised the description in the Fig. 2 legend as follows:
"(c) cryo-TOF-SIMS spectrum obtained from frozen, hydrated transverse surface of a lilac stem in the region containing the cambial zone and the differentiating xylem"

> Overall, the authors present meaningful data, but a more insightful discussion including the lignin composition (syringyl unit) of lilac stem and its distribution, how this knowledge can be applicable to other species, as well as some of the broader aspects of the research question would significantly strengthen this manuscript.

Thank you for the comment. We added a sentence on this section as follows:
"Intercellular transportation of lignin precursors has been discussed from the perspective of post-mortem lignification (Hosokawa et al., 2001; Pesquet et al., 2013). The increment of the syringyl to guaiacyl ratio of the lignin structural units in latter stage of angiosperm lignification (Terashima et al., 1986; Terashima and Fukushima, 1989; Fukushima and Terashima, 1990; Table S2) may be considered in discussions regarding the regulatory mechanisms of lignification in future studies."

For Table S2, we supplemented new data regarding lignin structural units via thioacidolysis and GC-MS measurements. Table S2 shows the S to G ratio of lignin structural units in phloem, differentiating xylem, and mature xylem region. Although this data suggesting the S-unit increment in latter stage of lignification is not new (as referred in the revised text), we can suggest that the characteristic syringin distribution in this study should be involved in such a mechanism.

Thank you for your many comments that will improve the manuscript. We tried as much as possible to clarify the discussion points and remove misleading statements. Now we believe that the manuscript is suitable for the next consideration.

==========

Reviewer: 2

Reviewer Report for the Authors
> This manuscript by Aoki et al. describes the distribution of the sinapyl alcohol glucoside in lilac stems. The study provides only limited novel knowledge in relation to previous work published by the same research group (Sci. Rep.

6: 31525 and Sci. Rep. 7: 5939). However, the authors have established an excellent tool to study plant biomolecules in a cell-specific manner. I would reconsider the manuscript, if they would be able to compare the methodology in species with direct biotechnological applications for the agri-food industry. The methodology has been already successfully proven in woody plants, the authors could perform a comparative study including legumes (e.g. soybean) and monocot (e.g. rice) plants. In fact, monocots are well-known to possess abundant fiber cells underneath the cortex that deposit more S-monolignol units. Some questions that could be addressed: are the S-units glucosides located in the same fiber cells? are they transported from other cells (phloem/xylem)? Any differences between these groups of plants or G- and S- glucosides? This is still an open question in the cell biology of lignification and would be an important study to advance the field.

Thank you for the positive and future-oriented comments. As the reviewer commented, it is quite important to do general discussion on the plant differentiation to find commonality within the plant kingdom. Considering this point, we would like to make modest claims for the following points:

The previous papers suggested by the reviewer use the same apparatus, but the target compound and plant species are completely different: one in Sci. Rep. 6 targets coniferin in gymnosperm and the other in Sci. Rep. 7 focuses on alkaloids in angiosperms. In this paper, we visualized syringin in angiosperms.

Second, as suggested in the introduction; the previous reports did not confirm the participation of syringin in lignification because of the peculiar seasonal changes and the syringin storage in phloem mainly. We believe that our present conclusion should be novel information pertaining to the role of syringin in plants.

As the reviewer mentioned, it should be better to expand the target plant species as a comparative study to extrapolate the findings. Nevertheless, we believe that the syringin distribution obtained in this present study will provide novel discussions regarding lignification and further plant physiological mechanisms concerning the intercellular transportation of biomolecules. Several questions the reviewer suggested at the end were very important but are difficult to thoroughly discuss based on the results of the study.

> To better reach a plant biology audience, I would recommend the authors to look at the transcriptional regulation of the molecules tracked. There is an excellent example of a transcriptomic study on poplar cross-sections (PNAS 98: 14732-14737) that would be really interesting to be addressed using these newly developed microscopic approaches.

Thank you for the suggestion. The transcriptomic imaging technique is quite important to discuss the driving force and the triggers for the mechanisms. The simultaneous spatiotemporal visualizations of gene expressions, enzyme activities, substrate quantities, cell development stages, and the co-existing compounds might be a good data set for the physiological mechanism discussions. We shall take the best efforts to obtain the proving and interconnected data sets in future study.

Finally, we would like to express our deep gratitude to the reviewer for the future-oriented comments. The manuscript was revised following the comments from the three reviewers. We believe that the manuscript is now suitable for the next consideration.
